# Universal Linear Fit Identification: A Method Independent of Data, Outliers and Noise Distribution Model and Free of Missing or Removed Data Imputation

**DOI:** 10.1371/journal.pone.0141486

**Published:** 2015-11-16

**Authors:** K. K. L. B. Adikaram, M. A. Hussein, M. Effenberger, T. Becker

**Affiliations:** 1 Research Group of Bio-Process Analysis Technology, Technische Universität München, Weihenstephaner Steig 20, 85354 Freising, Germany; 2 Institute for Agricultural Engineering and Animal Husbandry, Bavarian State Research Center for Agriculture, Vöttinger Straße 36, 85354 Freising, Germany; 3 Computer Unit, Faculty of Agriculture, University of Ruhuna, Mapalana, Kamburupitiy, Sri Lanka; 4 Lehrstuhl für Brau- und Getränketechnologie, Technische Universität München, Weihenstephaner Steig 20, 85354 Freising, Germany; University of California Berkeley, UNITED STATES

## Abstract

Data processing requires a robust linear fit identification method. In this paper, we introduce a non-parametric robust linear fit identification method for time series. The method uses an indicator *2/n* to identify linear fit, where *n* is number of terms in a series. The ratio *R*_*max*_ of *a*_*max*_ *− a*_*min*_ and *S*_*n*_ *− a*_*min*_ *n and that of *R*_*min*_ of *a*_*max*_ *− a*_*min*_ and *a*_*max*_ *n − S_*n*_ are always equal to *2/n*, where *a*_*max*_ is the maximum element, *a*_*min*_ is the minimum element and *S*_*n*_ is the sum of all elements. If any series expected to follow *y = c* consists of data that do not agree with *y = c* form, *R*_*max*_ *> 2/n* and *R*_*min*_ *> 2/n* imply that the maximum and minimum elements, respectively, do not agree with linear fit. We define threshold values for outliers and noise detection as *2/n* * (1 + *k*_*1*_ *)* and *2/n* * (1 + *k*_*2*_ *)*, respectively, where *k*_*1*_ > *k*_*2*_ and *0 ≤ k*_*1*_ *≤ n/2 − 1*. Given this relation and transformation technique, which transforms data into the form *y = c*, we show that removing all data that do not agree with linear fit is possible. Furthermore, the method is independent of the number of data points, missing data, removed data points and nature of distribution (Gaussian or non-Gaussian) of outliers, noise and clean data. These are major advantages over the existing linear fit methods. Since having a perfect linear relation between two variables in the real world is impossible, we used artificial data sets with extreme conditions to verify the method. The method detects the correct linear fit when the percentage of data agreeing with linear fit is less than 50%, and the deviation of data that do not agree with linear fit is very small, of the order of ±10^−4^%. The method results in incorrect detections only when numerical accuracy is insufficient in the calculation process.

## Introduction

Usage of parametric statistical methods to identify the behaviour of data is a topic for debate. In 1973, Francis Anscombe demonstrated that it is possible to have nearly identical statistical properties even with data sets that have considerable variation when graphed [1]. The four data sets used to show this phenomenon are known as Anscombe’s quartet [1]. Furthermore, Anscombe demonstrated the importance of the effect of outliers on statistical properties. Despite the distribution dissimilarities of the data sets of Anscombe’s quartet, the linear regression of all four data sets is the same. This implies that the statistical approach might not always identify the correct regression owing to the influence of outliers. There are four factors influencing regression detection: outliers and noise [2–6]; the nature of the distribution of the clean data, noise and outliers [7,8]; the number of outliers and the amount of noise [9–12] and missing data [13,14]. Of these four factors, three factors are related to outliers and noise.

In any domain, clean data are the data that follow the assumed data distribution model [15], while noise is the data that follow the assumed probability distribution [15,16]. Outliers are data that are not in agreement with the assumed clean data and noise models. In this paper, we consider the linear model *y = mx + c*, where *m* is the gradient and *c* is the intercept. When the aforementioned definition is applied to the linear model, clean data are the data that agree with the model. Noise can be defined as the data that are within a particular tolerance (e.g. ±x% from the correct value). Outliers are the data that agree with neither clean data nor noise. The most common approach is to remove outliers and noise with reference to the assumed models and then perform the regression analysis. Thus, outlier detection, noise detection and determination of regression of clean data are considered as separate, independent tasks. However, in our method, there is no separate regression analysis for locating linear fit. We first remove outliers and then remove noise using the same method, but with different weight parameters. Finally, the remaining data are the data that agree with linear fit.

Each outlier and noise detection method has a particular level of accuracy. Therefore, it is impossible to guarantee total outlier- and noise-free data. As a consequence, if the cleaned data still contain outliers and/or noise, the detected regression can be incorrect. The accuracy of the outlier detection and noise-removing methods depends on the distribution nature of the outliers and noise, the number of outliers and the amount of noise, which are dependent on the assumed data model. The most common model is the Gaussian distributions. Incorrect determination of the model will cause incorrect detection of outliers and noise. In other words, the accuracy of the selected method is totally dependent on the underlying models. Usually, outliers and noise have been removed, and regression is determined in accordance with the assumed regression model and remaining data that considered as clean data. The major drawback of this approach is that the determined regression is already affected by the influence of outliers and noise models.

The number of outliers and the amount of noise existing in a data set are critical factors when detecting outliers or noise. Especially when detecting outliers, their number plays a critical role. In addition, in a real-world data set, it is very common to have more than one outlier. Therefore, a robust outlier detection method must be capable of detecting multiple outliers. A large number of multiple outlier detection techniques have been proposed to accomplish this aim [17–19]. There are two phenomena, masking and swapping, that have a negative impact on the robustness of the outlier detection process [17]. Masking classifies detection of an outlier as a non-outlier, while swapping classifies detection of a non-outlier as an outlier.

As mentioned above, missing data imputation is another challenge. There are different methods for missing data imputation. These methods are also domain dependent, and there is no guarantee of accuracy when the data are not in accordance with the assumed models. The method we introduce in this paper is totally independent of missing data or removed data imputation.

Our method is based on the sum of the elements of a finite arithmetic progression (AP), which was introduced by Aryabhata (476–550 CE) [20,21]. Aryabhata was one of the greatest early mathematicians and astronomers [20,21] of India. In 499 CE, he introduced a method for calculating the sum of the elements of a finite AP or arithmetic sequence with *n* elements [22]. In 2014, we showed that Aryabhata’s equation for the sum of an AP can be used as a non-parametric method for detecting outliers in linear regression [23]. The method uses a single point as a reference point, and all detections are conducted with reference to this selected reference point. The method involves two steps, minimum-maximum-sum (MMS) and enhanced MMS (EMMS). MMS is used to remove all significant outliers one by one. Removing an outlier using MMS necessitates recalculating the entire series. After the removal of significant outliers, EMMS is used to remove non-significant outliers. EMMS uses a transformation technique before performing the detection of further outliers. MMS and EMMS are capable of locating outliers correctly when the reference point is not an outlier. When the reference point is an outlier, the method reports incorrect identifications of both outliers and non-outliers. This major drawback resulted in MMS and EMMS being unreliable for identifying outliers. Consequently, using MMS and EMMS jointly did not provide a reliable and robust method for determining linear fit.

Using an improved version of the same methodology, we were able to develop the method presented in this paper for determining linear fit. We expected outlier and noise detection and determination of regression to be possible using a single process that is independent of outlier, noise, data models and data imputation. In the existing literature, there is no such method for identifying a particular linear fit that is independent of models. In this paper, we introduce a single method that is capable of determining linear fit; removes outliers and noise; is independent of the distribution properties of clean data, outliers and noise; is independent of missing or removed data; is resistant to very high rates of outliers and noise (e.g. 50%) and yields no incorrect detections (masking or swapping). The method is suitable for time series or any data series that can be considered as or converted to time series. The most interesting feature of this method is that all five critical factors are addressed in one simple method with a very high level of accuracy. For this reason, we named it the Universal Linear Fit Identification (UniLiFI) method.

## Methodology

According to Aryabhata [22], the sum of the elements of an AP or arithmetic sequence with *n* elements is given by

$$S_{n}=\left(n/2\right)*\left(a_{1}+\;a_{2}\right),$$ (1)

where *a*_*1*_ is the first element, and *a*_*n*_ is the last element of the series.

Eq 1 has been used to achieve its original objective since its introduction. We have been unable to find direct applications of the original formula for other purposes. However, we have been able to use Eq 1 to locate outliers in linear regression [23].

An AP is a sequence of numbers (ascending, descending or constant) such that the difference between the successive terms is constant. The *n* ^*th*^ term of a finite AP with *n* elements is given by

$$a_{n}=d*\left(n-1\right)+\;a_{1},$$ (2)

where *d* is the common difference of successive members, and *a*_*1*_ is the first element of the series.

Eq 2 is a function of *n*, represents an AP and fulfils the requirements of a line (*y = mx + c*). A straight line is a series without outliers or noise (if there are outliers or noise, the series is not a line). Therefore, any arithmetic series that fulfils the requirements of an AP can be considered a series without outliers or noise.

Eq 1 can be represented as

$$2/n=(a_{1}+\;a_{n})/S_{n}\;;\;2/n\;\leq 1\;\;and\;\;2\;\leq n<\infty .$$ (3)

For any AP, the right-hand side (RHS) of Eq 3 is always *2/n*, which is independent of the terms of the series. In other words, if there are no outliers or noise, the value *(a*_*1*_ *+a*_*n*_ *)/S*_*n*_ will always equal 2*/n*. Therefore, the value 2/*n* can be used as a global indicator to identify any AP with outliers or noise. There are four facts in connection with Eq 3: 1. for any AP without outliers or noise, the value *(a*_*1*_ *+ a*_*n*_ *)/S*_*n*_ is always 2/*n*, which is independent of the terms of the series; 2. the converse of statement 1 is not always true (*i*.*e*. if the value *(a*_*1*_ *+ a*_*n*_ *)/S*_*n*_ is 2/*n*, this does not imply that the series is free of outliers or noise); 3. if the value *(a*_*1*_ *+ a*_*n*_ *)/S*_*n*_ is not 2/*n*, then the series always contains outliers or noise; 4. the converse of statement 3 is not always true (*i*.*e*. if there are outliers or noise, the value *(a*_*1*_ *+a*_*n*_ *)/S*_*n*_ is not always unequal to 2/*n*). However, there are still two situations that are always true (statements 1 and 3), enabling us to use Eq 1 for identifying outlier- and noise-free series. In real-world processes, it is impossible to have noise-free data series. Therefore, we ignore the relation in connection with statement 1 and use the relation in connection with statement 3.

Using statement 3, in 2014 we developed a two-step non-parametric method for identifying outliers in linear regression with reference to a single reference data point [23]. The two steps, MMS and EMMS, and their equations are shown as in Eqs 4 and 5, respectively.

If any series expected to follow *y = c* form consists of data that do not agree with *y = c* form,

$$MMS=\{\begin{array}{c} MMS_{max}=\;\frac{a_{max}\;-\;a_{min}}{S_{n}\;-\;a_{min}*n}=\;\{\begin{array}{c} >(2/n\;+\;w)\;;\\ \text{maximum is the outlier}\\ \leq \left(2/n\;+\;w\right);\;- \end{array}\\ MMS_{min}=\;\frac{a_{max}\;-\;a_{min}}{a_{max}*n-\;S_{n}}\;=\{\begin{array}{c} \leq \left(2/n\;+\;w\right);\;-\\ >\left(2/n\;+\;w\right)\;;\\ \text{minimum is the outlier} \end{array} \end{array}$$ (4)

where *a*_*max*_, *a*_*min*_, *S*_*n*_, *n*, and *w* are the maximum term of the series, the minimum term of the series, the sum of all terms of the series, the number of terms of the series, a weight where *0 ≤ w ≤ 1 − 2/n* and *R*_*w*_ = *2/n + w*, respectively.

$$EMMS=\{\begin{array}{c} EMMS_{max}\;=\;\frac{\left(a_{max}^{TT}-\;a_{min}^{TT}\right)}{\left(S_{n}^{TT}-\;a_{min}^{TT}*n\right)}=\;\{\begin{array}{c} >\left(2/n\;+\;w\right);\;\\ maximum\;is\;the\;outlier\\ \leq \left(2/n\;+\;w\right);\;- \end{array}\\ EMMS_{min}=\;\frac{\left(a_{max}^{TT}-\;a_{min}^{TT}\right)}{\left(a_{max}^{TT}*n-\;S_{n}^{TT}\right)}\;=\{\begin{array}{c} \leq \left(2/n\;+\;w\right);\;-\\ >\left(2/n\;+\;w\right);\;\\ minimum\;is\;the\;outlier \end{array} \end{array}$$ (5)

Where $a_{k}^{TT}=\left|a_{k}^{T}-{\text{x}}_{k}*\left(Ga^{T}/\;Gx\right)\right|$, $\;a_{k}^{T}=\;a_{k}–\;a_{0}$ *x*_*k*_ is the index of data, *a*_*k*_ is the *k* ^*th*^ term of the series, *k* = 0, 1, …, *n −* 1, *n* is the number of elements in the current window, $Ga^{T}\;=\;\overset{n-1}{\underset{k\;=\;0}{\sum }}a_{k}^{T}$, $Gx\;=\;\overset{n-1}{\underset{k=0}{\sum }}X_{k}$, $S_{n}^{TT}=\overset{n-1}{\underset{k=0}{\sum }}a_{k}^{TT}<>0$, *2/n + w = R*_*w*_, and *w* is the weight, *0 ≤ w ≤ 1 − 2/n*.

In the abovementioned method, the first value (*a*_*0*_) is used as the reference point. Therefore, the method gives correct detections when the first point is not an outlier. Furthermore, MMS is used for removing significant outliers, while EMMS is used for removing non-significant outliers. When using MMS, it is possible to obtain incorrect detections of outliers as the result of selecting a small value for *w* [23]. The recalculation process in MMS and the transformation used in EMMS provide correct transformations only when the reference point is not an outlier [23]. However, it is impossible to determine the nature of a point in advance.

After considering all drawbacks, we introduced a new method based on the same principle. The new method contains a new transformation technique using multiple reference points, shown in Eq 6. The number of reference points can be in the interval [1, *n*], where *n* is the total number of data points in the selected data set. However, the process uses each reference point separately as the reference point and transforms the data with

$$a_{k\;|\;r}^{TT}=\{\begin{array}{c} a_{k\;|\;r}^{T}\;–\left(x_{k\;|\;r}^{T}\text{ * }\left(Ga_{k\;|\;r}^{T}/Gx_{k\;|\;r}^{T}\right)\right),\;\;\;\;\;\;\;x_{k}\;-x_{r}\geq 0\\ -\left(a_{k\;|\;r}^{T}\;–\left(x_{k\;|\;r}^{T}\text{ * }\left(Ga_{k\;|\;r}^{T}/Gx_{k\;|\;r}^{T}\right)\right)\right),\;\;x_{k}\;-x_{r}<0 \end{array}$$ (6)

Where $\;a_{k\;|\;r}^{TT}$ is the *k* ^*th*^ item of the transformed series with reference to the reference point *r*, $a_{k\;|\;r}^{T}\;=\;a_{k}\;–\;a_{r}$,$x_{k\;|\;r}^{T}=\;x_{k}\;–\;x_{r}$, *x*_*k*_ is the index of data, *a*_*k*_ is the *k* ^*th*^ term of the series, (*x*_*r*_, *a*_*r*_) is the reference point, *k* = 0, 1, …, *r*, …, *n −* 1, *r* = 0, 1, …, *n −* 1, *n* is the number of elements in the current window, *r* is the index of the reference data point, $Ga_{k\;|\;r}^{T}\;=\;\overset{n-1}{\underset{k\;=\;0}{\sum }}a_{k\;|\;r}^{T}$ and $Gx_{k\;|\;r}^{T}\;=\;\overset{n-1}{\underset{k\;=\;0}{\sum }}x_{k\;|\;r}^{T}$.

The transformation in Eq 6 can convert all data to the form *y = c* if there are no outliers or noise. If the data set consists of outliers or noise, the transformed data do not agree with the form *y = c* and *MMS* can locate the outliers or noise. The form *y = c* is independent of the occurrence sequence of the data [23]. Therefore, there is no effect from missing or removed data on the outlier detection process. In addition, *w* of Eq 5 can be expressed as *w* = 2 * k / *n*, where 0 < *k* ≤ (*n* /2) − 1 [23]. If *MMS*(*D*)_max|*r*_ refers to *MMS*_*max*_ with reference to reference point *r* for data set D and if *MMS*(*D*)_min|*r*_ refers to *MMS*_*min*_ with reference to reference point *r* for data set D. Then, Eq 7 provides the application of MMS on *a* ^*TT*^.

$$MMS\left(a^{TT}\right)=\{\begin{array}{c} MMS{\left(a^{TT}\right)}_{\text{max}|\;r}=\;\frac{\left(a_{\text{max}|\;r}^{TT}-\;a_{\text{min}|\;r}^{TT}\right)}{\left(S_{n|\;r}^{TT}-\;a_{\text{min}|\;r}^{TT}*n\right)}\;=\;\{\begin{array}{c} >2/n*\left(1\;+\;k\right);\\ maximum\;is\;the\;outlier\\ \leq 2/n*\left(1\;+\;k\right);\;- \end{array}\\ MMS{\left(a^{TT}\right)}_{\text{min}|\;r}=\;\frac{\left(a_{\text{max}|\;r}^{TT}-\;a_{\text{min}|\;r}^{TT}\right)}{\left(a_{\text{max}|\;r}^{TT}*n-\;S_{n|\;r}^{TT}\right)}\;=\{\begin{array}{c} \leq 2/n*\left(1\;+\;k\right);\;-\\ >2/n*\left(1\;+\;k\right);\\ minimum\;is\;the\;outlier \end{array} \end{array}$$ (7)

where $S_{n|r}^{TT}=\;\overset{n-1}{\underset{k=0}{\sum }}a_{k}^{TT}$ , $S_{n|r}^{TT}-\;a_{min}^{TT}*n<>0$, and $a_{max}^{TT}*n-\;S_{n|r}^{TT}<>0$.

After transformation, outliers or noise are detected using Eq 7. If an outlier or noise is detected, it is removed from both transformed and original data sets. Then, the transformation is applied again, and outlier detection is performed until one of the termination conditions is reached. In general, there are three termination conditions: 1. $a_{\text{max}|\;r}^{TT}\;=\;a_{\text{min}|\;r}^{TT}\;=\;0$; 2. a selected reference point is detected as an outlier; or 3. no more outliers are detected.

Table 1 and S1 File show a complete process cycle for achieving a candidate data set for linear fit, with reference to the second item of the series.

**Table 1:** A complete process circle for achieving a candidate data set for linear fit with reference to the second item (30) of the data set. Detection process must be conducted considering each term as a reference point. However, in this example shows calculations only with reference to the second item. In the first iteration *MMS(a* ^*TT*^ *)*_*max|*2_ > *2/n* and fulfils the detection condition. Thus, in the first iteration $a_{\text{max}|2}^{TT}$ is the term that not agrees with the linear fit. Therefore, (8, 41.81) was removed and excluded from the calculations in second iteration. This process was continued until the termination condition ($a_{\text{max}|\;2}^{TT}\;=\;0$ and $a_{\text{min}|\;2}^{TT}\;=\;0$) is reached in fourth iteration. Note that in this example, *k = 0* and *r = 2*. Also, see S1 File for better understanding on the calculation process.

		Iteration 1			Iteration 2			Iteration 3			Iteration 4		
*X*	*a*	$x_{k\;|2}^{T}$	$a_{k\;|2}^{T}$	$a_{k\;|2}^{TT}$	$x_{k\;|2}^{T}$	$a_{k\;|2}^{T}$	$a_{k\;|2}^{TT}$	$x_{k\;|2}^{T}$	$a_{k\;|2}^{T}$	$a_{k\;|2}^{TT}$	$x_{k\;|2}^{T}$	$a_{k\;|2}^{T}$	$a_{k\;|2}^{TT}$
6*^2^	22*^2^	-1	-8.000	-2.42	-1	-8.000	-2.25	-	-	-	-	-	-
7	30**	0	0.000	0.00	0	0.000	0.00	0	0.000	0.0E+0	0	0	0
8*^1^	41.81*^1^	1	11.810	1.39	-	-	-	-	-	-	-	-	-
9*^3^	50.001*^3^	2	20.001	-0.85	2	20.001	-0.50	2	20.001	7.8E-4	-	-	-
10	60	3	30.000	-1.27	3	30.000	-0.75	3	30.000	-3.3E-4	3	30	0.0
11	70	4	40.000	-1.69	4	40.000	-1.00	4	40.000	-4.4E-4	4	40	0.0
	Sum	9	93.811		8	82.001		9	90.001		7	70	
$a_{\text{max}|2}^{TT}$				1.39^‡^			0.00			7.8E-4^‡^			0.0
$a_{\text{min}|2}^{TT}$				-2.42			-2.25^‡^			-4.4E-4			0.0
*n*				6			5			5			4.0
$S_{n|2}^{TT}$				-4.85			-4.50			0.00			0.0
*R*_*k*_ = *2/n*				0.33			0.40			0.40			0.5
*MMS(a* ^*TT*^ *)*_*max|2*_				0.39	*(>0*.*33)*		0.33			0.55	*(>0*.*40)*		-
*MMS(a* ^*TT*^ *)*_*min|2*_				0.29			0.50	*(>0*.*40)*		0.31			-

Legend:

**: Reference data point.

^‡^: Term identified as the outlier in the relevant iteration.

*^x^: Removed in the relevant iteration and not considered for the next iteration.

At the end of the process cycle with reference to a particular reference data point, the remaining data set is a candidate data set for linear fit. This process is applied for all selected reference points and yields a candidate data set for linear fit with reference to each reference point. Then, for each candidate data set, the linear correlation is calculated using

$$r_{xy}=\frac{n\sum x_{i}y_{i}-\;\sum x_{i}\sum y_{i}}{\sqrt{n\sum x_{i}^{2}-{\left(\sum x_{i}\right)}^{2}}\sqrt{n\sum y_{i}^{2}-{\left(\sum y_{i}\right)}^{2}}},$$ (8)

where *x* is the independent variable and *y* is the dependent variable.

A correlation with 1 ≥ |*r*_*xy*_| ≥ 0.8, 0.6 ≥|*r*_*xy*_| > 0.8, 0.3 ≥|*r*_*xy*_| > 0.6 or 0.0 ≥|*r*_*xy*_| > 0.3 is generally described as very strong, moderately strong, fair or poor correlation [24], respectively. The abovementioned intervals are true for the linear relations that have *y = mx + c* form, only when *m ≠ 0*, where *c* is a constant. When *m = 0*, the linear relation is of the form *y = c*. However, when *y = c*, *n*Σ*x*_*i*_ *y*_*i*_ − Σ*x*_*i*_Σ*y*_*i*_ = 0 (numerator of (Eq 8)) and $n\sum x_{i}^{2}-{\left(\sum x_{i}\right)}^{2}\;=\;0$ (part of denominator of Eq 8), *r*_*xy*_ becomes undefined. This situation prevents identification of linear fits that have the form *y = c*. Therefore, when Σ*x*_*i*_ *y*_*i*_-Σ*x*_*i*_Σ*y*_*i*_ = 0 and $n\sum x_{i}^{2}-{\left(\sum x_{i}\right)}^{2}\;=\;0$, we stipulate that *r*_*xy*_ = 1.

**Fig 1:**
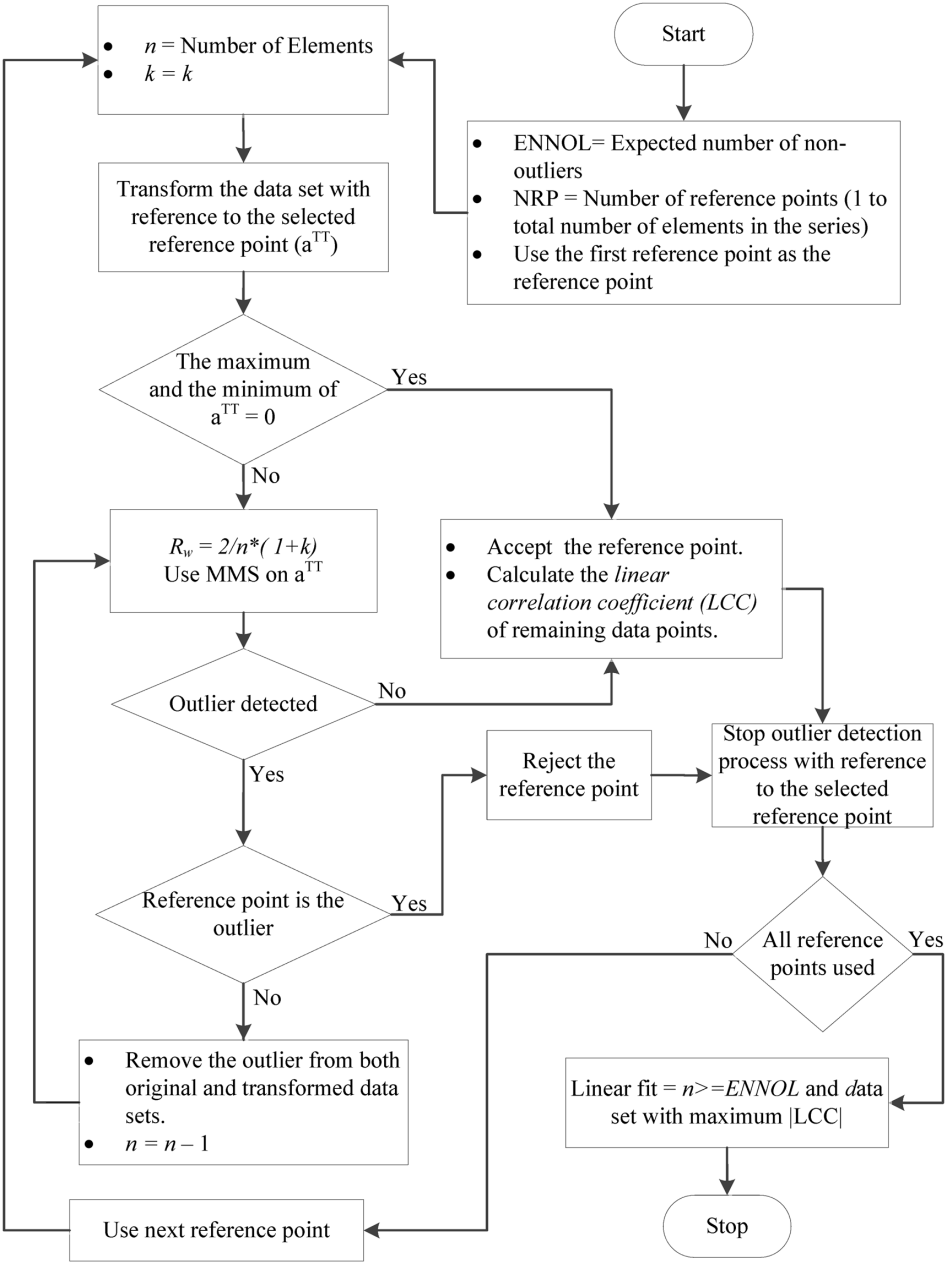
The first method of applying the new multiple reference point linear fit algorithm. When terminating conditions are fulfilled with reference to a particular reference point, outlier detection is terminated. Then, the process continues with the next reference point until all reference points are finished. Among the different candidate linear fits in relation to different successful reference points, the best linear fit is determined by considering the linear correlation coefficient and the number of data points.

**Fig 2:**
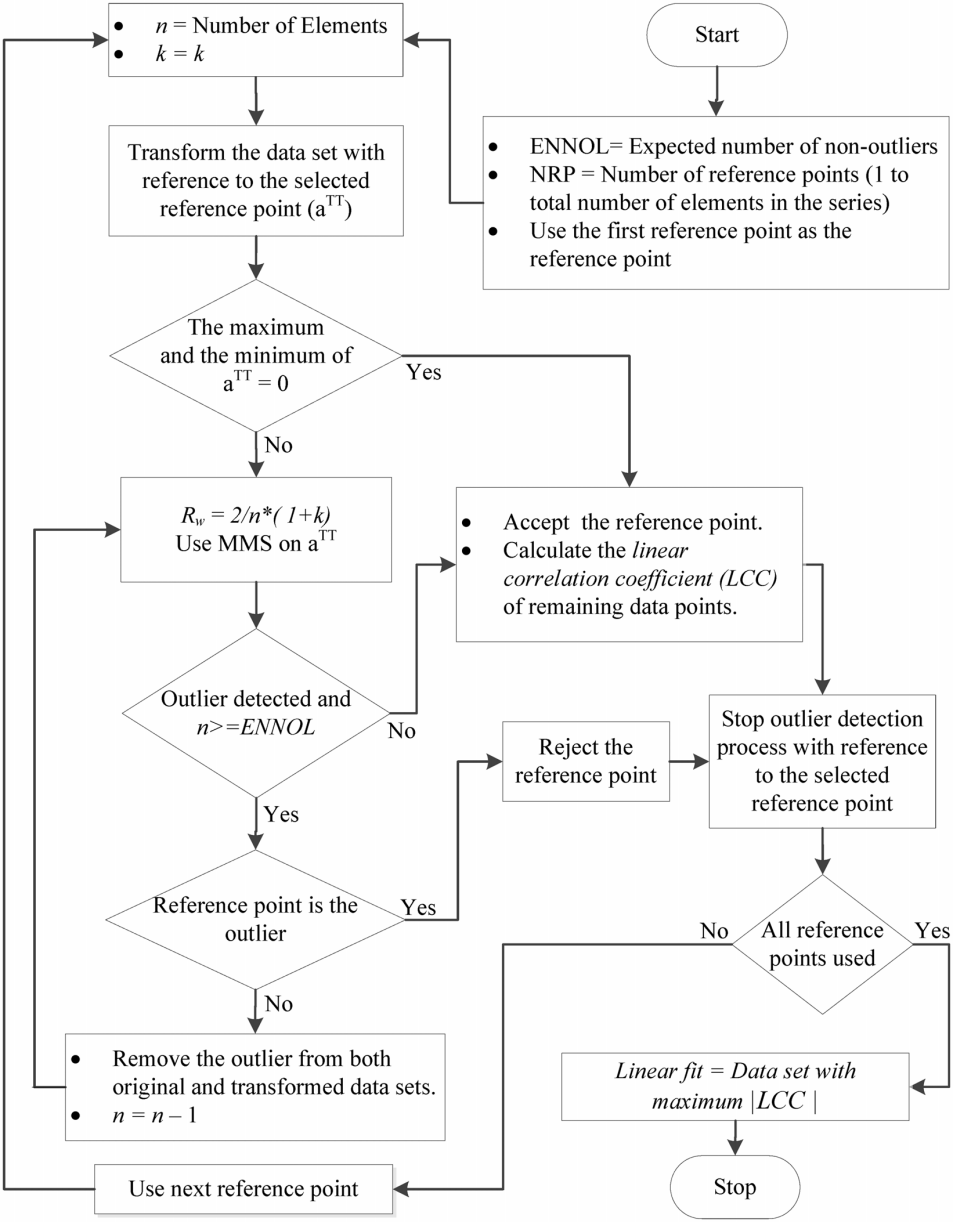
The second method of applying the new multiple reference point linear fit algorithm. In this method, the expected number of non-outliers (ENNOL) is used as a termination condition. When terminating conditions are fulfilled with reference to a particular reference point, outlier detection is terminated. Then, the process continues with the next reference point until all reference points are finished. Among the different candidate linear fits in relation to different successful reference points, the best linear fit is determined by considering the linear correlation coefficient.

When considering the accuracy of Eq 8, a data set with fewer points satisfying the equation will provide better correlation than the best resultant linear fit data set leading to a wrong decision. For example, a data set with two points will give the best correlation (|*r*_*xy*_| = 1) despite the best fitting. Therefore, we define a minimum number of data points that must be in the final linear fit. The best linear fit is defined as the data set with the maximum absolute correlation (|*r*_*xy*_|) and the minimum number of data points. These two criteria can be used in different ways to determine the best linear fit depending on the requirements. The decision diagrams elaborated in Figs 1 and 2 express two different implementation methods of the new multiple reference point linear fit algorithm.

**Fig 3:**
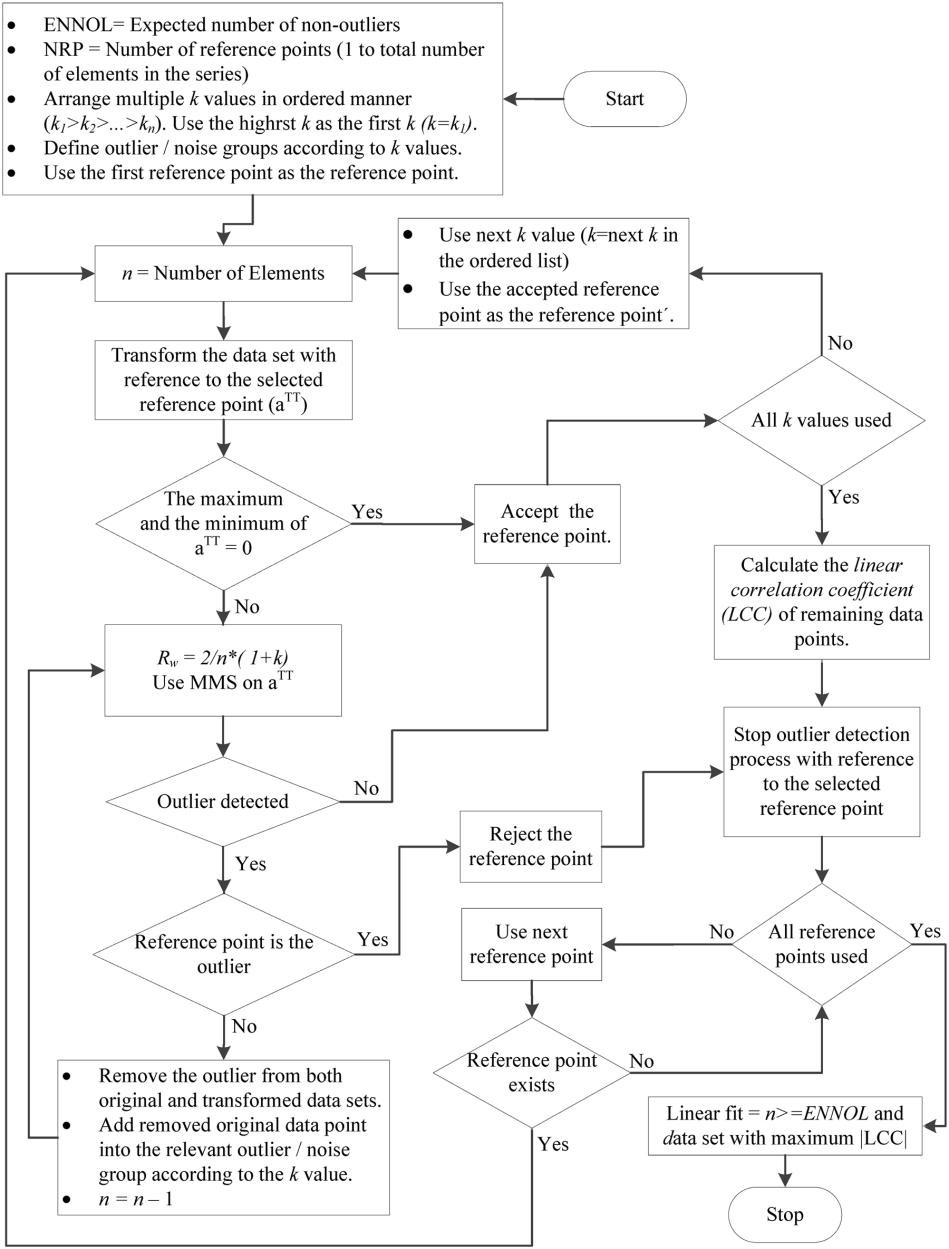
Improved version of the first method shown Fig 1 for grouping outliers or noise into several groups based on different *k* values. The method shown in Fig 2 can also be improved for grouping outliers or noise into several groups in the same manner.

Using this method, the data points that do not agree with linear fit can be categorized into several categories using different *R*_*k*_ values based on different *k* values, in several steps. After identifying different *k* values, the data with the highest *k* (or highest *R*_*k*_) value are first checked, and then the cleaned data are used as input for the next step with the next highest *k* value. Fig 3 elaborates the implementation of the multi-step multiple reference linear fit algorithm, based on the first method elaborated in Fig 1. The second method elaborated in Fig 2 can be improved for locating linear fit while grouping data that do not agree with linear fit using the same technique.

To check the best linear fit, the algorithm was tested using several synthetic and real data sets based on zero-based numbering (the first term of a series is assigned the index 0). Among the artificial data sets, the first three data sets of Anscombe’s quartet [1] can be considered time series. The real data were from biogas plants and were automatically recorded with a frequency of 12 data points per day (*i*.*e*. every other hour) over a period of seven months. With real data, it is impossible to find a perfect linear relation between two variables. Nevertheless, among the different parameters, we selected the NH_4_^+^ content measured in g/kg of fresh matter, which we expected to maintain linear behaviour during stable operation. We selected seven segments of different sizes for evaluating the algorithm. In some data sets, there were initial missing elements. Performance of the new method was evaluated using a linear regression model and MMS/EMMS.

## Results and Discussion

**Fig 4:**
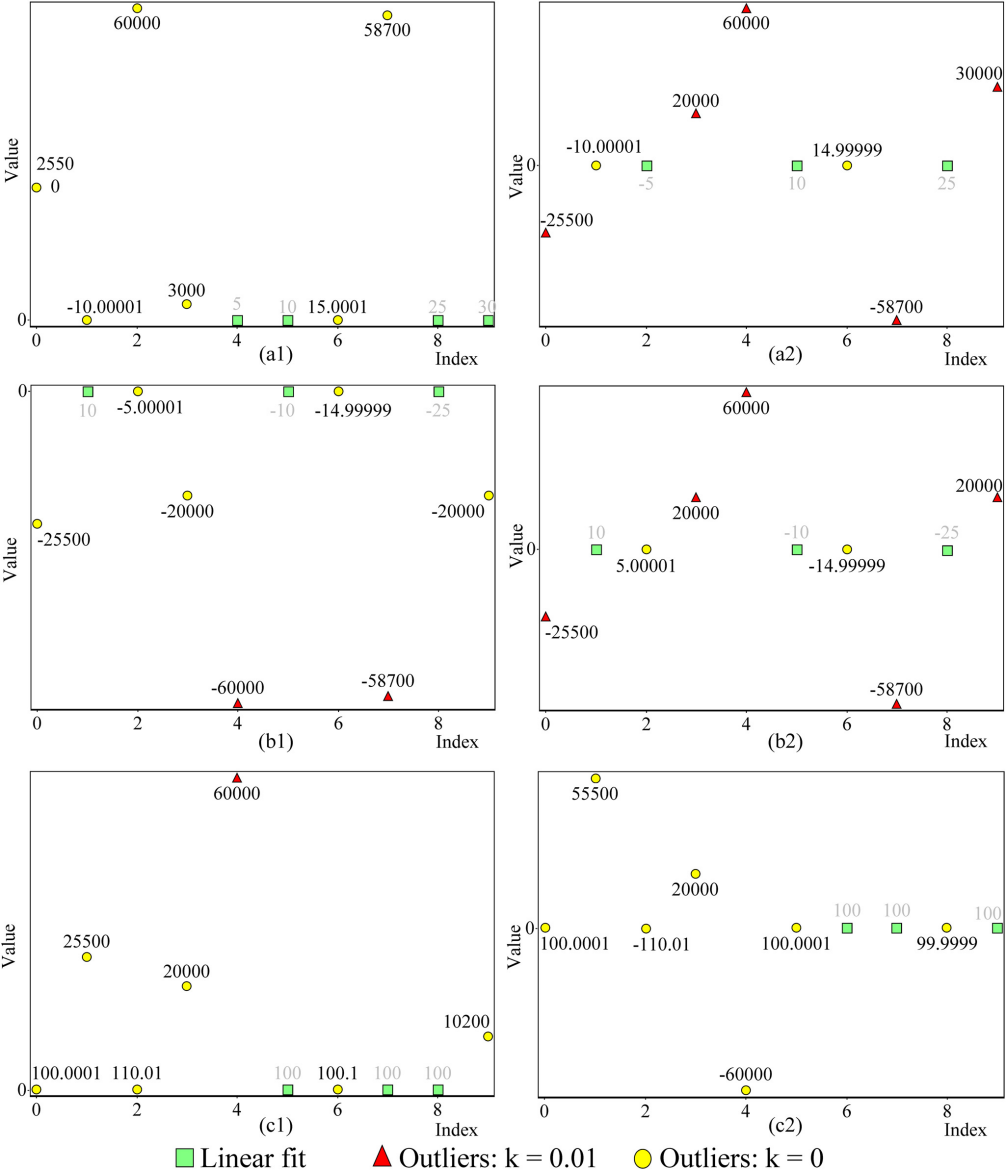
The gradient of linear fits shown in (a1) and (a2), (b1) and (b2) and (c1) and (c2) are ascending, descending and constant, respectively. In data sets (a1), (b1) and (c1), all data points that do not agree with linear fit are located on one side (non-Gaussian) of linear fit. In data sets (a2), (b2) and (c2), all data points that do not agree with linear fit are located on both sides of linear fit. In all data sets, fewer than 50% of the data points agree with linear fit. Some of the data not agreeing with linear fit deviate more than ±10^4^ from the correct value. At the same time, there are data points that have very small deviation, as small as ±10^−4^, from the correct value. Whatever the condition, the new method was capable of identifying robust linear fit. In all plots, the reference point is the first data point in linear fit, which was automatically detected during the detection process (all the points were considered as the reference point). For data set of plots in this figure see S2 File. Fig 5 consists of three data sets of Anscombe’s quartet [1], which can be considered as APs. As shown in Fig 5, the new method was capable of identifying the nearest data set that agrees with linear fit. We set the number of minimum data points at five for all examples in Fig 5. In Fig 5(c) and 5(d) represent the third data set of Anscombe’s quartet and use different *k* values. When the *k* value changes, the reference point and number of non-outliers are not the same for the same ENNOL. Furthermore, no masking or swapping occurred in relation to any *k* value we used for linear fit identification.

We used synthetic data sets with different sizes (4 to 1,000 points) and real data sets for evaluating our new linear fit identification method. Here, we include some data sets with extreme conditions. Fig 4 shows six data sets, each consisting of 10 data points, and the data sets that agreed with linear fit have either a positive gradient, a negative gradient or a constant value. In all data sets, fewer than 50% of the data points agreed with linear fit. Some of the data not in agreement with linear fit deviated more than ±10^4^ from the correct value. At the same time, there are data points that have very small deviation, as small as ±10^−4^, from the correct value. All data points that did not agree with linear fit in data sets (a1), (b1) and (c1) shown in Fig 4 are located on one side (non-Gaussian) of linear fit. Whatever the condition, the new method was capable of identifying a robust linear fit.

**Fig 5:**
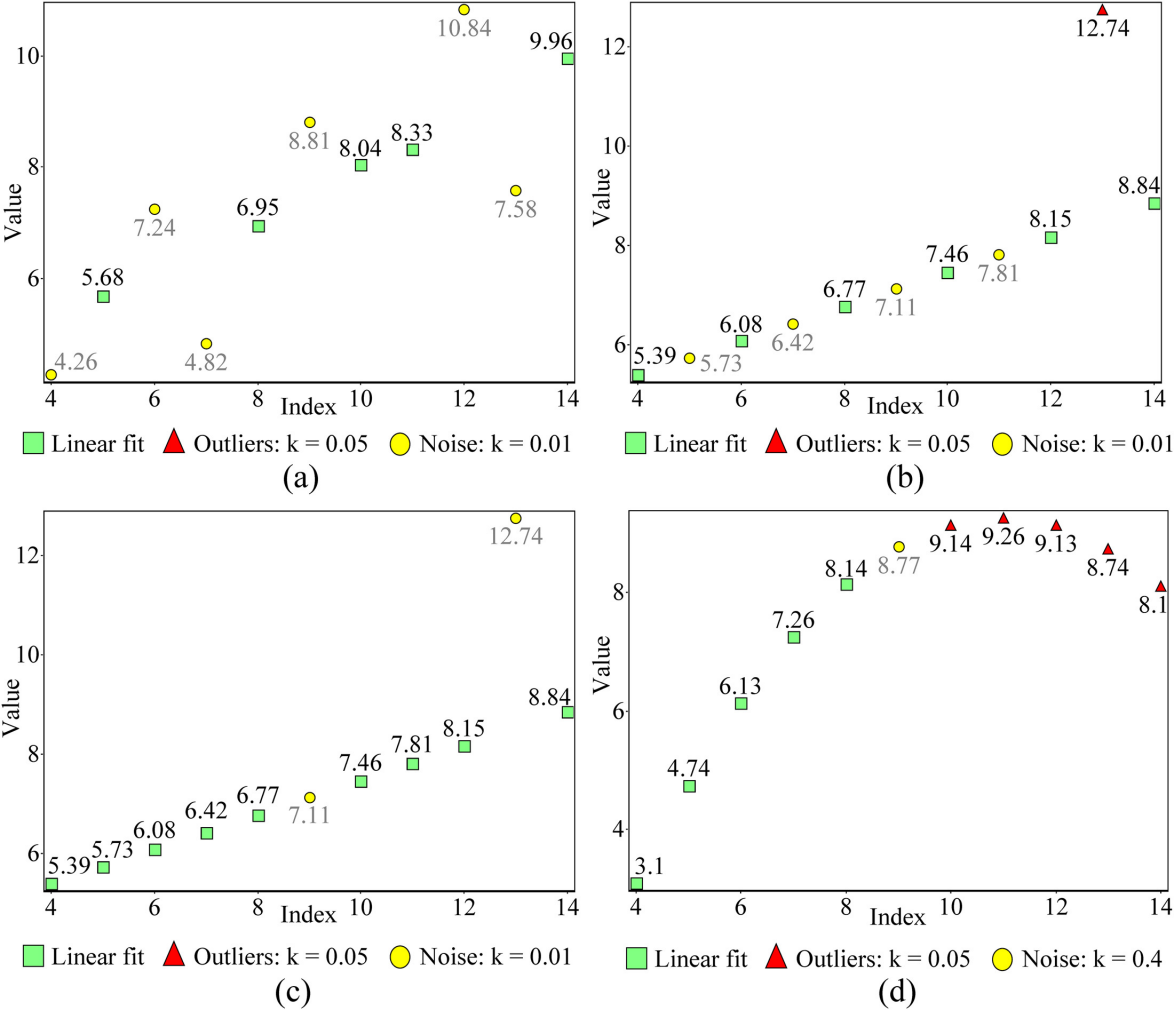
Plots (a) and (b) show the first and second data set of Anscombe’s quartet and used the same value of *k*. Plots (c) and (d) represent the third data set of Anscombe’s quartet and used different *k* values. In all detections, ENNOL was set to five. When the *k* value changes, the reference point and number of points in linear fit are not the same for the same ENNOL (Plots (c) and (d)). In all plots, the reference point (the first term of the linear fit) was automatically detected during the detection process (all the points were considered as the reference point). For data set of plots in this figure see S3 File.

**Fig 6:**
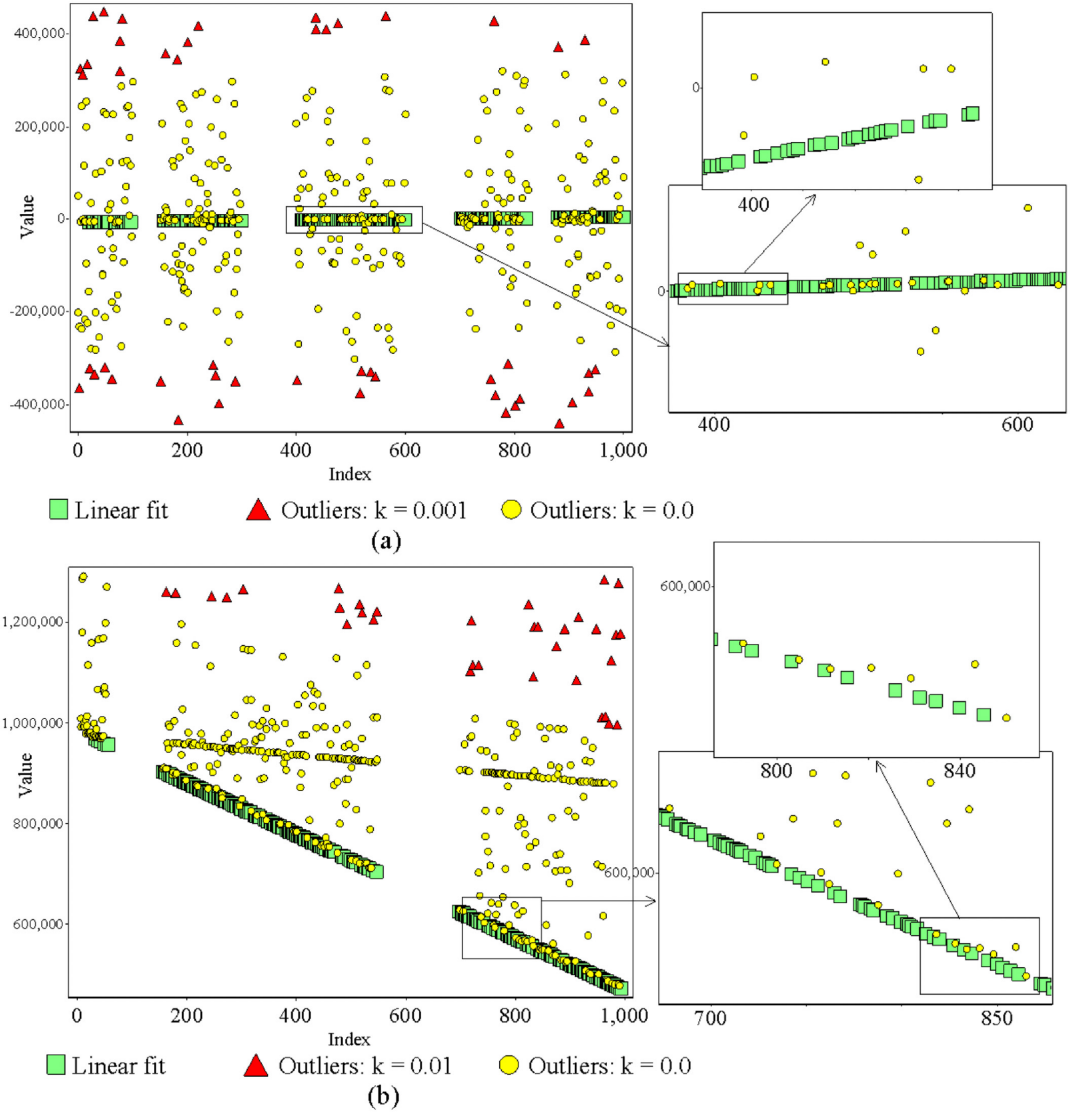
Plots (a) and (b) show two artificial data sets, each consisting of a data set with 100% agreement with unknown linear regression. The number of data points agreeing with linear fit is less than 50% of total existing data points. In plot (a), data points that do not agree with linear fit lie on both sides of linear fit and exhibit four initial missing data regions of 50, 100, 100 and 50 data points (total 300 initial missing data). In plot (b), data points that do not agree with linear fit are located on one side of linear fit and exhibit two initial missing data regions of 100 and 150 data points (total 250 initial missing data). In both plots, data points that do not agree with linear fit are in the range of ±10^−2^ to ±10^4^. Though both data sets represent very extreme conditions, the method was capable of locating all data points that agreed with linear fit without swapping or masking. Zoomed areas of selected areas that contain very near values to linear fit demonstrate the ability of the proposed method. In plots (a) and (b) the reference points (the first term of the linear fit) were automatically detected during the detection process as 20 and 26, respectively (all the points were considered as the reference point). For data set of plots in this figure see S4 File.

The new method showed its ability to identify linear fit with large window sizes as well. Fig 6 shows two data sets consisting of 1,000 data points, each with less than 50% of the data points agreeing with exact linear fit (regression is unknown). The data points that do not agree with linear fit are in the range of ±10^−2^ to ±10^4^. In Fig 6, plot (a) bears four initial missing data regions, with 50, 100, 100 and 50 data points (total 300 initial missing data), while plot (b) bears a total of 250 initial missing data, with 100 and 150 missing data regions. In Fig 6 plot (a), the data that do not agree with linear fit lie on both sides of linear fit, while in plot (b), all data points that do not agree with linear fit lie on one side of linear fit.

**Fig 7:**
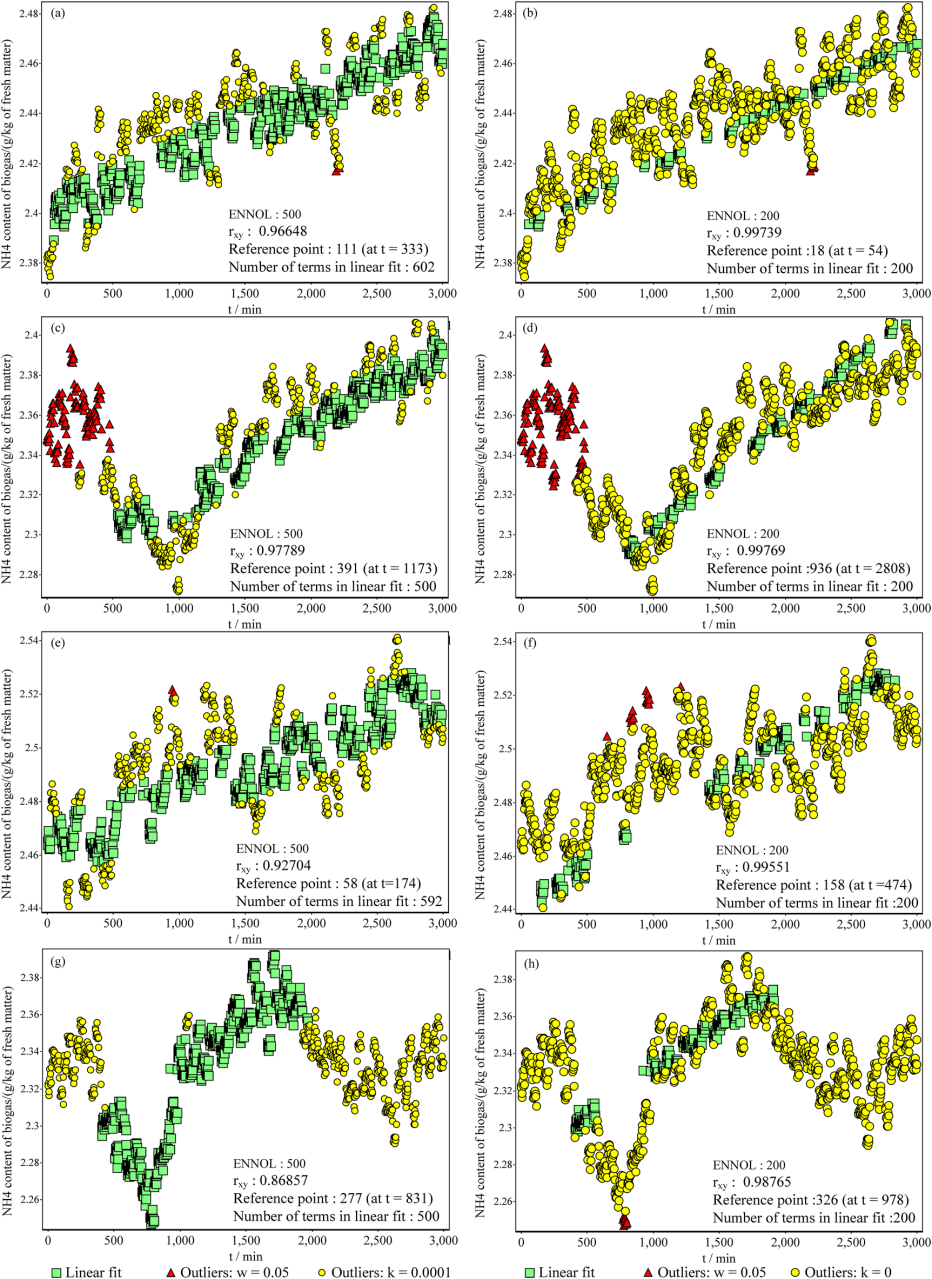
Plots show four selected windows of data captured automatically from a biogas plant in a three-minute interval (each window consists of 1,000 data points). The left side shows the linear fit detection in relation to a particular criterion, while the right side shows the linear fit detection of the relevant left-side data set in relation to narrower criteria than the left-side plot. In all cases, the method identified the most suitable linear fit in relation to the selected window. When the criteria are narrowed, the detection is sharp and there is a sub-set of the linear fit identified in relation to wider criteria. In all plots, the reference point (the first term of the linear fit) was automatically detected during the detection process (all the points were considered as the reference point). For data set of plots in this figure see S5 File.

All mentioned properties above are very extreme conditions. However, the new method identified linear fit with a high level of accuracy. Furthermore, in Fig 6 plot (b), there is a set of data that have a nearly linear relation and makes the situation more extreme. All results prove that the new method is capable of locating all data points that agree with linear fit without masking or swapping. This accuracy cannot be achieved with a conventional least squares method or with MMS/EMMS. Nevertheless, when the deviation of the value of a data point was less than ±10^−2^ from its correct value, sometimes we observed 0.5% swapping and masking with the new method. However, this is very rare situation and not the result of a failure of the method but of the limited numerical accuracy of the programming language (Visual C++ 2010) [25]. This is more visible when the number of data points is large and their deviation is very small. Therefore, we recommend using a programming platform with high numerical accuracy for better performance with the new method. Fig 7 shows eight data windows of data captured automatically from a biogas plant. Each window consists of 1,000 data points, with results included in relation to two different conditions. The left side of Fig 7 shows identified linear fits of four different windows. The right side of Fig 7 consists of linear fits of four windows corresponding to those shown on the left side with narrower linear fit identification criteria than on the left side. When considering all eight situations, in plots (a1) and (c1), *R*_*k*_ reaches its limit before ENNOL. Furthermore, in all situations, *r*_*xy*_ is greater than 0.8 and implies a very strong linear fit [24]. However, plots on the RHS, which have narrower criteria, showed higher correlation than the corresponding plot on the left side. As in the artificial data sets, with these actual data, there is no swapping or masking. This is a major advantage of this method over any other method. The linear fits in relation to the first data set (plots (a1) and (a2)) do not show any exceptionality and no resistance to acceptance. In contrast, in the second data set (plots (b1) and (b2)), there is a minimum that clearly shows two potential regions for linear fit. On the other hand, in the third data set (plots (c1) and (c2)), there are two regions based on the data density.

In both data sets, the new method was able to locate the best linear fit, which can be identified even visually. In the second data set, the new method omitted one potential area and identified linear fit from the longer half. However, in the third data set, the identified linear fit is from both regions. This shows the ability of the new method to identify the best linear fit without influence from data density and other data in the considered window. In the fourth data set, there are a minimum and a maximum that clearly show three potential linear fit regions. Furthermore, region 3 has the highest data density. However, the new method was able to identify linear fit from region 2, which has low data density. Again, this confirms the previous observation. Plots (c2) and (d2) show another feature of the new method: the identified linear fits clearly consist of two segments separated by a no-data area.

According to the most popular least squares method, data points that agree with linear fit are the data points around the trend line. However, our aim is to identify the most potential data sets that agree with the linear fit. Therefore, that detection of least squares method cannot be considered as good detection according to our requirement. Therefore, the abilities of the new method are in a better position when identifying linear fit because it is capable of identifying the best fit among the several positive candidate linear fits. This type of detection can be performed using an appropriate mask. Sometimes it is necessary to use several masks for identifying different types of linear fit, such as one mask for identifying linear fits with positive gradients, one for identifying linear fits with negative gradients and one for identifying linear fits that are constant. In contrast, the new method is capable of identifying any type of linear relation. Therefore, the new method can be considered as very useful for identifying linear fit.

When considering the theoretical environment of the equations used in the method, there are several situations that must be addressed. Theoretically, there are two situations for which the method could become invalid. The first situation occurs when $Gx_{k\;|\;r}^{T}\;=\;0$ in Eq 6. To overcome this situation, we propose a solution that can be used in normal situations as well. The proposed method in this paper always suspects the maximum and minimum as the data points that do not agree with linear fit. If the suspected data points were removed, it is possible to have better approximation for the gradient as well. However, after removing both suspected values, it is still possible to have the same situation. Therefore, as a standard, when $Gx_{k\;|\;r}^{T}\;=\;0$, removing one suspected point will guarantee the prevention of an undefined situation. In addition, if no undefined situation arises, it is better to exclude both suspected points. As we mentioned earlier, this technique can be applied throughout the process. However, this requires additional computational effort. We used the same technique to improve the outlier detection power of Grubb’s test and obtained significant improvement.

The second invalid situation occurs when $\;S_{n}^{TT}-\;a_{min}^{TT}*n\;=\;0$ or $a_{max}^{TT}*n-\;S_{n}^{TT}=0$. Then, according to (7), $a_{\text{max}|\;r}^{TT}\;=\;a_{\text{min}|\;r}^{TT}$ (the maximum and minimum of the transformed series are the same). In addition, the transformed value of the considered reference point is always zero. Therefore, $S_{n}^{TT}-\;a_{min}^{TT}*n\;=\;0$ or $a_{max}^{TT}*n-\;S_{n}^{TT}\;=\;0$ represents the status in which all values of the transformed series are zero. This state also represents a totally outlier and noise free series and is a termination condition.

In addition to the two abovementioned undefined situations, there is another situation in which it is not possible to determine the termination point. The situation in which all remaining terms agree with linear fit, with $MMS{\left(a^{TT}\right)}_{\text{max}|\;r}\;=\;MMS{\left(a^{TT}\right)}_{\text{min}|\;r}$, can be considered as the termination point of Eq 7. However, there can be a very rare situation that is in disagreement with the normal situation. For example, if the transformed series is 0, −1.1, −2.1, 2.2, 1, 0.3, then $MMS{\left(a^{TT}\right)}_{\text{max}|\;0}\;=\;MMS{\left(a^{TT}\right)}_{\text{min}|\;0}$ occurs (both values are equal to 0.33). In this case, the transformed series does not agree with linear fit, even though it satisfies the termination condition. Therefore, it is necessary to verify that the situation is a real termination situation. One possible remedy for overcoming this situation is to recalculate the data series by temporarily excluding one data point that is not a suspected point and is not equal to zero. If the same situation still occurs even after removing the data point, it can be considered as the real termination point. Otherwise, conduct the calculation without temporarily removing the term, and add it in the next iteration. If the transformed series 1.1, 1.1, 0, 0, 0, −1.1, −1.1 again satisfies $MMS{\left(a^{TT}\right)}_{\text{max}|\;0}\;=\;MMS{\left(a^{TT}\right)}_{\text{min}|\;0}$, then the aforementioned method cannot be used. Therefore, the only possible solution is to consider all non-zero terms as terms that do not agree with linear fit.

## Conclusions and Outlook

The new method shows very promising results in the area of linear fit identification. The method is non-parametric and capable of identifying all data points that agree with linear fit without swapping or masking. A particular strength of the new method is that it detects the most probable linear fit in the selected window despite the influence of data density, missing data, removed elements, percentage of data agreeing with linear fit and manner of distribution of data points. In other words, the introduced method can be considered as a universal method for linear fit identification. In this paper, we focused on identifying a single linear fit. However, the method could be enhanced for identifying multiple linear fits in the selected window.

## Supporting Information

S1 File Example calculation: A complete process circle for achieving a candidate data set for linear fit with reference to a certain reference point. (XLSX)

S2 File Data sets of all the plots in Fig 4. (XLSX)

S3 File Data sets of all the plots in Fig 5. (XLSX)

S4 File Data sets of all the plots in Fig 6. (XLSX)

S5 File Data sets of all the plots in Fig 7. (XLSX)
